# The *Waddlia* Genome: A Window into Chlamydial Biology

**DOI:** 10.1371/journal.pone.0010890

**Published:** 2010-05-28

**Authors:** Claire Bertelli, François Collyn, Antony Croxatto, Christian Rückert, Adam Polkinghorne, Carole Kebbi-Beghdadi, Alexander Goesmann, Lloyd Vaughan, Gilbert Greub

**Affiliations:** 1 Center for Research on Intracellular Bacteria, Institute of Microbiology, University Hospital Center and University of Lausanne, Lausanne, Switzerland; 2 Center for Biotechnology, Bielefeld University, Bielefeld, Germany; 3 Institute of Health and Biomedical Innovation, Queensland University of Technology, Brisbane, Australia; 4 Institute of Veterinary Pathology, Vetsuisse Faculty, University of Zurich, Zurich, Switzerland; University of Hyderabad, India

## Abstract

Growing evidence suggests that a novel member of the *Chlamydiales* order, *Waddlia chondrophila,* is a potential agent of miscarriage in humans and abortion in ruminants. Due to the lack of genetic tools to manipulate chlamydia, genomic analysis is proving to be the most incisive tool in stimulating investigations into the biology of these obligate intracellular bacteria. 454/Roche and Solexa/Illumina technologies were thus used to sequence and assemble *de novo* the full genome of the first representative of the *Waddliaceae* family, *W. chondrophila*. The bacteria possesses a 2′116′312bp chromosome and a 15′593 bp low-copy number plasmid that might integrate into the bacterial chromosome. The *Waddlia* genome displays numerous repeated sequences indicating different genome dynamics from classical chlamydia which almost completely lack repetitive elements. Moreover, *W. chondrophila* exhibits many virulence factors also present in classical chlamydia, including a functional type III secretion system, but also a large complement of specific factors for resistance to host or environmental stresses. Large families of outer membrane proteins were identified indicating that these highly immunogenic proteins are not *Chlamydiaceae* specific and might have been present in their last common ancestor. Enhanced metabolic capability for the synthesis of nucleotides, amino acids, lipids and other co-factors suggests that the common ancestor of the modern *Chlamydiales* may have been less dependent on their eukaryotic host. The fine-detailed analysis of biosynthetic pathways brings us closer to possibly developing a synthetic medium to grow *W. chondrophila*, a critical step in the development of genetic tools. As a whole, the availability of the *W. chondrophila* genome opens new possibilities in *Chlamydiales* research, providing new insights into the evolution of members of the order *Chlamydiales* and the biology of the *Waddliaceae*.

## Introduction

The *Chlamydiaceae* were long considered a phylogenetically isolated group of closely-related bacteria. However, during the past decades, the order *Chlamydiales* has been enriched by the discovery of five additional families: *Criblamydiaceae*, *Parachlamydiaceae*, *Rhabdochlamydiaceae, Simkaniaceae*, and *Waddliaceae* [1], [2], [3], [4]. Members of these families are commonly called *Chlamydia*-related bacteria due to their phylogenetic relationship to *Chlamydiaceae* and their *Chlamydia*-like cycle of replication with two developmental stages; the elementary body (EB) or infectious particle, and the reticulate body (RB) or replicative form.

Globally, members of the *Chlamydiaceae* family are recognized as a widespread and clinically significant cause of disease in humans and animals. It comes as no surprise that evidence is emerging to support a pathogenic role for *Chlamydia*-related bacteria as well [5]. *Simkania negevensis* and *Parachlamydia acanthamoebae* are suspected to cause respiratory tract infections [6], [7]. Moreover, *P. acanthamoebae* was recently shown to be associated with abortion in cows [8].

*Waddliaceae* might also cause bovine abortion since two strains of *W. chondrophila* have been isolated independently from aborted bovine fetuses [9], [10] and a serological study in cows supported an abortigenic role [11]. More recently, a prospective study demonstrated an association between miscarriage and the presence of anti-*Waddlia* antibodies in humans [12]. Moreover, DNA of *W. chondrophila* was detected in one respiratory sample of a patient with community-acquired pneumonia [13] and in samples taken from children with bronchiolitis [14], suggesting that, as previously described for *Coxiella burnetii*, this emerging agent of miscarriage may also cause respiratory tract infections. The pathogenic potential of *W. chondrophila* is further indicated by its rapid growth within human macrophages [15].

The advent of genomics was a fundamental step in the characterization of obligate intracellular bacteria such as the *Chlamydiaceae,* which are widely recognized pathogens. Since 1998, the release of fourteen complete genome sequences from members of the *Chlamydiaceae*, ranging in size from 1Mb to 1.2Mb, provided major advances in the study of their biology and the identification of virulence factors [16]. The genome analysis revealed a high overall similarity in gene content and gene order between the various *Chlamydiaceae*, although regions of gene rearrangement, referred to as the “plasticity zone”, can be found near the terminus of replication [17]. Incomplete pathways for tricarboxylic acid cycle or biosynthesis of key amino acids, nucleotides or cofactors revealed a strong dependency on host-derived metabolites [16]. Insights into bacterial virulence were provided by the discovery of a complete type three secretion system (T3SS) [18] and conserved virulence factors such as CPAF [19]. Furthermore, periplasmic and outer membrane proteins unique to the *Chlamydiaceae* were discovered and compose a highly disulphide crosslinked matrix that supply the structural resilience usually provided by the peptidoglycan layer in most Gram-negative bacteria [20]. These include the abundant cysteine-rich proteins OmcA and OmcB as well as the major components of the chlamydial outer membrane complex, the beta-barrel porins OmpA and PorB [16]. Moreover, a chlamydial specific family of autotransporters, the highly diverse polymorphic outer membrane proteins (pmps), has been implicated in adhesion and in the host immune response [21], [22]. Most of these cell wall proteins are highly immunogenic and are used for serological diagnosis or vaccine development [23], [24].

Among the *Chamydia-*related bacteria, sequences from only two strains belonging both to the *Parachlamydiaceae* family have been released to date. *P. acanthamoebae* Hall's coccus was published as an unfinished genome of 3Mb in a combined proteomics and genomics approach [25] whereas the environmental *Protochlamydia amoebophila* UWE25 was fully sequenced and exhibited a 2.4Mb genome [26], i.e. approximately twice the size of classical chlamydia. *P. amoebophila* showed limited conservation of genome structure together with the presence of several repetitive elements [26]. Moreover, its chromosome contains a 100 kb-long genomic island encoding a potentially functional F-like DNA conjugative system [27] and more than 70 leucine-rich repeat proteins [28]. Although the bacterium displayed improved biosynthetic abilities as compared to *Chlamydia* species, a similar dependency on host derived metabolites was observed [26]. Intriguingly, no homologs to major outer membrane proteins and polymorphic membrane proteins have been identified, suggesting these highly immunogenic proteins might be *Chlamydiaceae* family specific.

The availability of genome sequences within new families of the *Chlamydiales* order is crucial to better understand the biology of *Chlamydia*-related bacteria. Therefore, we sequenced the full genome of *W. chondrophila* using both 454/Roche and Solexa/Illumina technologies, also uncovering the presence of a low copy number plasmid. The genome annotation revealed numerous intriguing features presented here which we anticipate will help stimulate and drive further research into this fascinating and medically important bacterial order.

## Results

### General genome features

**Figure 1:**
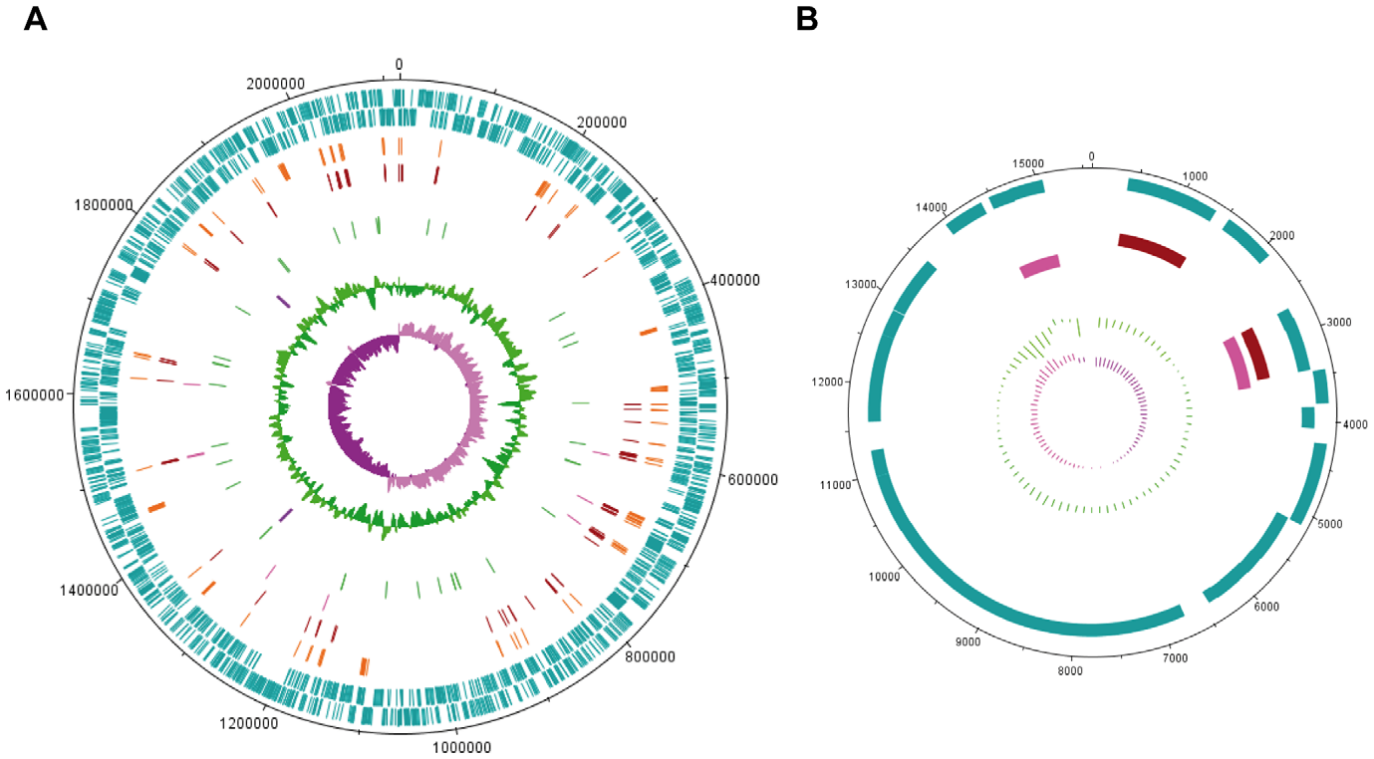
*Waddlia* chondrophila genome. Circular representation of *Waddlia chondrophila* chromosome (A) and plasmid (B). From the outermost circle, circles 1 and 2 show the plus- and minus-strand ORFs (blue). Circles 3 and 4 exhibit the repeated sequences (>200 bp, in orange) and the tranposases/integrases (red), respectively. Circle 5 represents the homologous regions between the plasmid and the chromosome (pink), whereas circles 6 and 7 show the location of tRNAs (green) and rRNAs (dark purple), respectively. Finally, the two innermost circles (8 and 9) show the GC content (dark green) and GC skew (purple). Lanes 3, 6 and 7 are absent in the plasmid representation.

The genome of *Waddlia chondrophila* WSU 86-1044 consists of a circular chromosome of 2′116′312 bp with a G+C content of 43.8% and a 15′593 bp circular plasmid with a G+C content of 37.6% (**Figure 1** and **Table 1**). The chromosome sequence displays a typical “V”-inverted shape on a cumulative GC skew plot (**Figure S1**), allowing the origin and terminus of replication to be located and the assembly accuracy to be confirmed [29], [30]. Another commonly used marker for the origin of replication is *dnaA* [31]. However, like other *Chlamydiales*, *W. chondrophila* encodes two copies of *dnaA*, none of which is linked to the minimum of the cumulative GC skew (**Figure S1**). Using bioinformatics analyses, a large number of repetitive sequences (>200 bp) were identified encompassing 4.9% of the chromosome, a significantly higher proportion than other sequenced *Chlamydiales* (**Figure 1** and **Table 1**).

**Table 1:** Main characteristics of *Chlamydiales* genomes.

	*Chlamydia trachomatis* D/UW-3/CX	*Chlamydophila pneumoniae* CWL029	*Waddlia chondrophila WSU 86-1044*	*Protochlamydia amoebophila* UWE25
Genome size	1′042′519	1′230′230	2′116′324	2′414′465
GC content	41%	40%	44%	34%
% coding	89%	88%	92%	82%
Nb of protein coding genes	895	1122	1934	2031
Nb of tRNAs	37	38	37	35
Nb of rRNA operons	2	1	2	3
% repeats	0.04%	0.7%	4.9%	1.5%
Plasmid size	7′493	–	15′593	–

Main characteristics of representative genomes from three families within the *Chlamydiales* order, as extracted from NCBI genome database (*Chlamydiaceae* and *Parachlamydiaceae*) or directly from the genome sequence (*Waddliaceae*). “% repeats” includes all repetitions larger than 200 bp, excluding rRNAs regions when present in several copies.

Two sets of rRNAs and 37 tRNA genes were identified as well as 1934 protein coding genes, which represent 92% of the whole genome. A putative function or family membership could be inferred for 1243 (65%) of them, whereas 253 (13%) are conserved hypothetical proteins and the remaining 438 (23%) show no similarity to known proteins (**Table S1** and **Figure S2**). Of the conserved hypothetical proteins, the major group of 156 proteins is most similar to hypothetical proteins from the *Parachlamydiaceae* family. The remainder show best BLAST hits against Eukaryotes (2), Archaea (4), *Chlamydiaceae* (4) and various other bacterial phyla (87). As expected, all essential components for DNA replication, transcription and RNA translation were successfully identified and mostly belong to the core set of *Chlamydiales* genes. The comparison of *W. chondrophila* encoded proteins with those of *C. trachomatis* and *P. amoebophila* showed the large proportion of family-specific proteins and proteins poorly conserved at the amino acid level within this highly diverse bacterial order (**Figure S3**). The X-plot representation of conserved genes highlighted small collinear regions between *Waddlia* and *Protochlamydia*, even though numerous rearrangements occurred, changing gene orientation and position. In contrast, conserved gene order is hardly distinguishable between *Waddlia* and *Chlamydia.*

### *Waddlia chondrophila* plasmid

The comparison between the average read depth of the *W. chondrophila* chromosome (40x) and its plasmid (440x) indicates that the plasmid is present in about 11 copies per cell. It encodes 22 proteins that mostly show no homology to other chlamydial plasmid proteins, with the exception of an integrase that exhibits 54% identity to the plasmid integrase pCpA1_003 of *C. psittaci*. The *Waddlia* chromosome contains numerous small regions (16–24 bp) identical to sequences in the plasmid. In addition, 7 chromosomal regions ranging from 57 bp to 849bp that encode entire or partial integrated transposases share between 99% and 100% identity with the two plasmid transposases (**Figure 1**). Each of these two transposases is strongly similar to several proteins encoded on the *P. amoebophila* genome. Finally, two adjacent genes were found to be integrated into the *Waddlia* chromosome sharing 88% nucleic acid identity to their plasmid counterparts. One of the encoded proteins is homologous to MazF, an endoribonuclease of the MazEF module, one of the most thoroughly studied toxin-antitoxin systems [32], that might be involved in the stable maintenance of the plasmid during cell division. Normally, *mazE* encodes a labile antitoxin that prevents the lethal effect of the stable toxin encoded by *mazF*. Although the neighbouring genes of *mazF* do not show any sequence similarity to *mazE*, one of them may represent the necessary antitoxin.

### Virulence factors and resistance to environmental stresses

**Figure 2:**
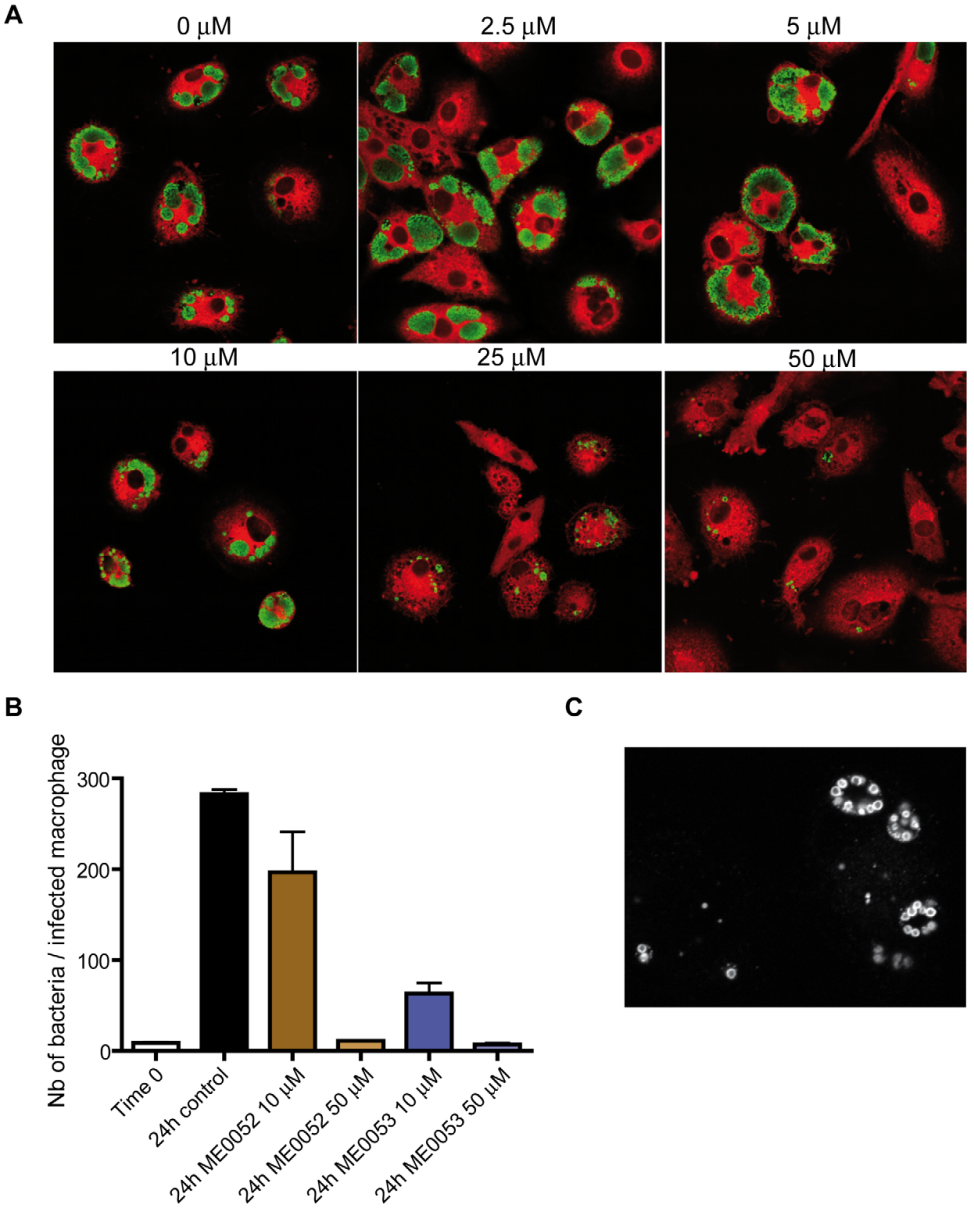
Dose-dependent inhibitory effects of the T3SS inhibitors ME0052 and ME0053 on *Waddlia chondrophila* infection in human macrophages. (**A**) Immunofluorescence staining of *Waddlia chondrophila* (green) in human macrophages (red) after treatment with ME0052 (see Methods S1). The dose-dependent growth inhibition was assayed using ME0052 at concentrations ranging from 0 to 50 µM. (**B**) Dose-dependent inhibitory effects quantified by immunofluorescence. The effect of T3SS inhibitors on the growth of *W. chondrophila* in human macrophages was demonstrated by counting the number of bacteria per infected macrophage. (**C**) Interestingly, replicating reticulate bodies are observed in close association with the inclusion membrane as shown in this confocal image taken 8h post-infection. Such a close association of the bacteria with the inclusion membrane may facilitate T3SS-mediated translocation of effectors to the host cytosol. This bacterial localization is in accordance with a model proposed by Peters *et al*. in 2007 suggesting that a tight contact of the bacteria with the inclusion membrane is required for chlamydial replication and differentiation.

As frequently described for intracellular bacteria, *W. chondrophila* encodes a T3SS devoted to inoculation of bacterial effectors into the host cytoplasm. Clusters of genes encoding the T3SS are spread over the bacterial chromosome as previously shown for other *Chlamydiales* (**Figure S4**) [18], [33]. The functionality and the requirement of the T3SS for *Waddlia* survival and replication in human macrophages were demonstrated by the effective inhibition of bacterial growth with T3SS specific inhibitors [34] (**Figure 2**). Interestingly, several genes encoding homologs to SycE and SycD chaperones are located adjacent to genes encoding hypothetical proteins that could represent putative T3SS effectors (**Figure S4**).

Several systems specific to *Waddlia* are likely involved in resistance to professional phagocytes such as amoebae and/or macrophages where the bacterium is able to escape lysosomal degradation and to grow rapidly [15], [35]. The *mrp* and *trk* systems as well as a putative carbonate permease and a carbonic anhydrase are probably involved in pH homeostasis by importing protons. Furthermore, homologs to superoxide dismutase and catalase as well as a nitric oxide reductase are likely involved in defense against radical oxygen species and nitrous oxides.

To resist environmental stresses, *W. chondrophila* possesses at least four heavy metal exporters for copper, zinc, cadmium and a possible tellurite reductase/permease, that could be involved in defense against toxic metals. Moreover, the bacterium encodes several multidrug efflux pumps of the RND family that may confer resistance to detergents, lipophilic drugs or bile salt derivatives.

A putative class-C β-lactamase might be responsible for the previously described *in vitro* resistance of *W. chondrophila* to ampicillin and ceftriaxone [36]. A putative peptidase S66 family protein and a putative undecaprenyl-diphosphatase 1 also indicate a possible resistance to microcin and bacitracin. Finally, several antibiotic resistance mechanisms were identified with the presence of proteins related to multidrug resistance MarC and MATE families suggesting that the bacteria could present a large pattern of resistance.

### Host parasitism and bacterial metabolism

*Waddlia* genome analysis revealed a degree of host independence, compared with other members of the *Chlamydiales* order, with the ability to produce energy independently from its host through oxidative phosphorylation. Reduced cofactors issued from complete TCA cycle and glycolysis are funneled along the electron transport chain to produce ATP. As in *P. amoebophila,* the presence of a F_O_F_1_ ATP synthase complex, in addition to a V_1_V_0_ ATPase complex conserved in the *Chlamydiaceae*, enhances its energy production capacity and improves its adaptability in energy-depleted environments. Furthermore, *W. chondrophila* contains the enzymatic components of the glyoxylate bypass enabling the utilization of fatty acids or acetate, in the form of acetyl-CoA, as a carbon source.

**Figure 3:**
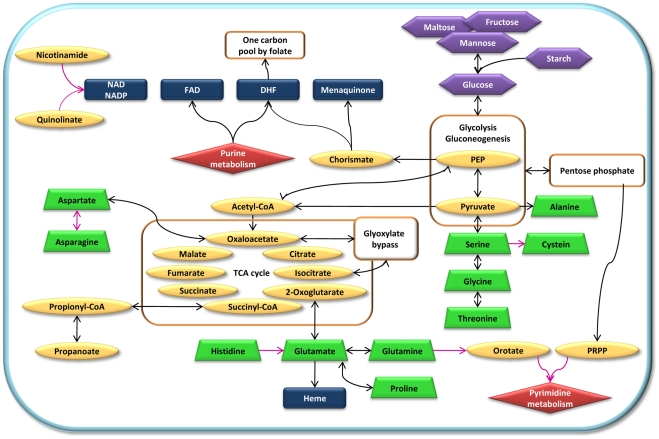
Major metabolic pathways of *Waddlia chondrophila*. Schematic representation of the major metabolic pathways identified in the genome of *Waddlia chondrophila*. Main metabolic pathways and intermediary components are shown in orange and yellow. Amino acids are represented in green, vitamins and cofactors in blue, nucleotides in red and sugars in purple. Pathways present in *Waddlia chondrophila* but not in other *Chlamydiales* are highlighted with pink arrows.

As summarized in **Figure 3**, the bacterium displays enhanced anabolic capabilities for key molecules such as cofactors, nucleotides or amino acids. No homolog to *P. amoebophila* NAD+/ADP transporter (*ntt_4*) [37] was found, but enhanced biosynthetic abilities indicate that *W. chondrophila* likely synthesizes NAD from an intermediary metabolite such as quinolinate or nicotinamide imported through another system. Five nucleotide transporters similar to *ntt_1*, *2* and *3* of *P. amoebophila* potentially enable the import of all nucleotides [37], [38]. Despite the presence of genes for nucleotide parasitism and unlike other *Chlamydiales*, *W. chondrophila* possesses all enzymes to convert L-glutamine in UMP and all pyrimidine derivatives necessary for replication and transcription (**Figure S5**). In contrast, a complete purine biosynthesis pathway could not be reconstructed but an active purine conversion, that is not present in other members of the *Chlamydiales* order, was identified (**Figure S5**).

*W. chondrophila* harbors the genetic material to produce at least ten of the twenty classical amino acids (**Table S2**). This bacterium completely lacks genes for the biosynthesis of tryptophan that are at least partially present in other *Chlamydia* and seems unable to produce tyrosine and phenylalanine but, instead, encodes five transporters devoted to general or specific aromatic amino acid import. Furthermore, many oligo-peptides and amino acid transporters or permeases have been identified and can likely import a variety of amino acids from the environmental medium.

Lipid metabolism also exhibits interesting features with the presence of additional enzymes for glycerophospholipid, glycerolipid and sphingolipid metabolism compared to other *Chlamydiales*. More interestingly, unlike *P. amoebophila* and *C. trachomatis*, *W. chondrophila* possesses a complete operon encoding the mevalonate pathway in the biosynthesis of isoprenoids precursors, whereas only one gene could be identified in the non-mevalonate pathway used by both *P. amoebophila* and *C. trachomatis*.

### Bacterial cell wall

The chlamydial cell wall differs from that of the majority of extracellular Gram-negative bacteria, the classical protective peptidoglycan being replaced by a highly disulphide-linked proteinaceous layer in the infectious EB [39] . Upon entering the cell, the EB is released from the constraints imposed by its protective corsett by reducing the disulphide-linked network of proteins, allowing it to swell in size as the replicating body forms. This process must be tightly regulated and *W. chondrophila* possesses several conserved periplasmic chlamydial redox enzymes. Major components of the proteinaceous network are OmcA and OmcB, a highly diverse family of polymorphic outer membrane proteins (pmp) and the outer membrane protein (omp) beta-barrel porins OmpA and PorB. A striking feature of these porins are conserved cysteine rich clusters of CxCxC or CxxC or CC or CxxCxxC signature sequences, essential in the covalent cross-linking of the periplasmic Omc proteins and the outer membrane proteins [40], [41].

**Figure 4:**
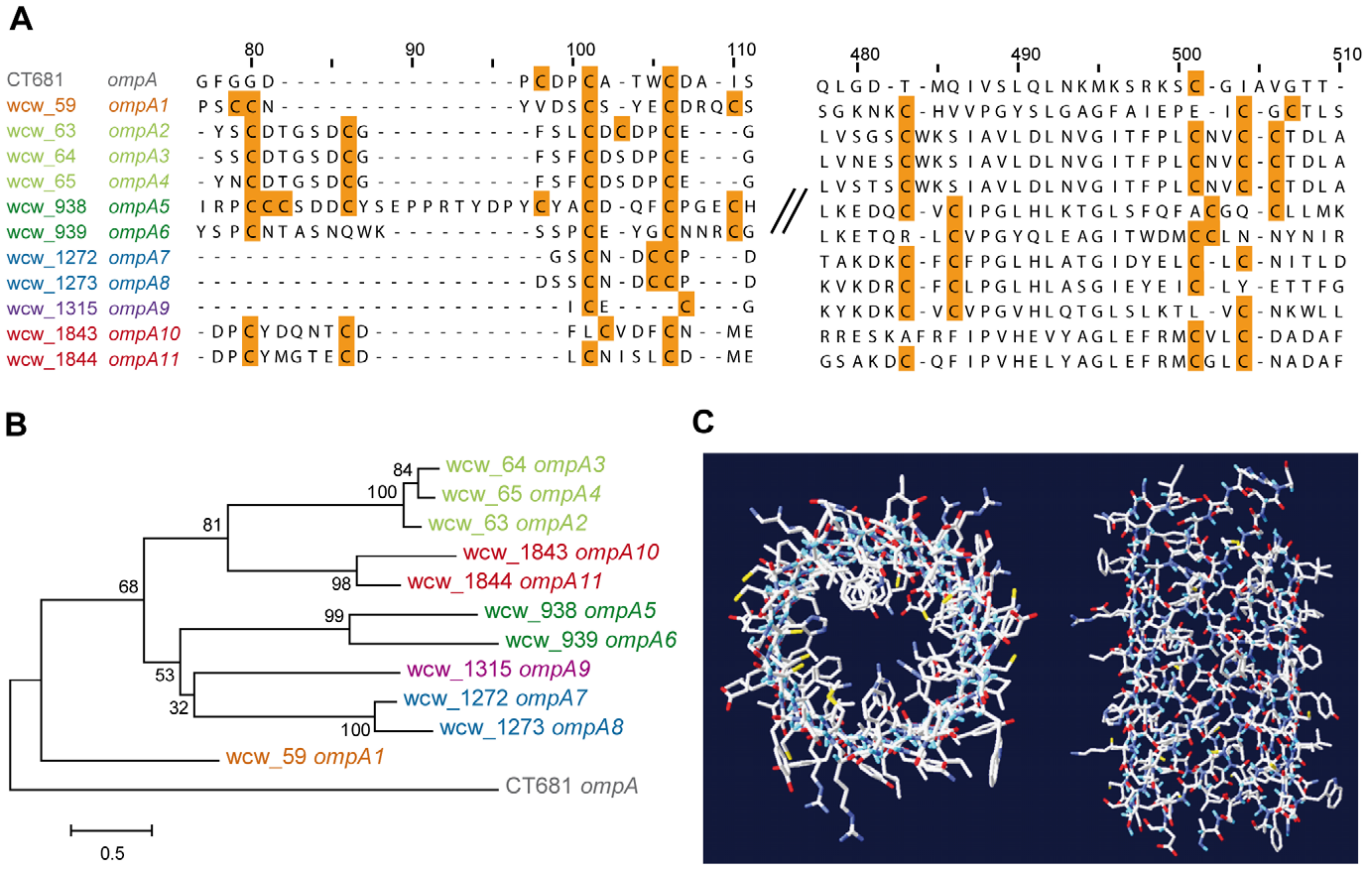
OmpA family proteins: alignment, tree and structure. Examples of conserved cysteine clusters (**A**) and the corresponding bootstrapped neighbor-joining tree (**B**) of the eleven members of the *Waddlia chondrophila* OmpA family relative to *Chlamydia trachomatis* OmpA. Whereas 8–9 conserved cysteines are present in OmpA and PorB of the *Chlamydiaceae*, the *Waddlia* OmpA family is even more richly endowed, with between 13 and 19 cysteines present. Genes are colored according to their clustering on the genome sequence. (**C**) Structure prediction of OmpA10 with the program partifold and using a small hydrophobic beta-barrel as template. The image on the left is viewed from above the plane of the membrane, showing the barrel pore, the image on the right is viewed from the side showing the membrane spanning barrel. The exact structures, including the number of membrane spanning beta strands, remain speculative until they can be anchored by biochemical data.

Most impressive in *W. chondrophila*, is a novel OMP family of 11 putative beta-barrel proteins or porins with C-rich signatures, partially shared with the *Chlamydiaceae* (**Figure 4**). In addition, conserved motives and structural analyses revealed the presence of a putative autotransporter protein that shows similarity to a gene in the *P. amoebophila* genome (**Figure S6**). These proteins might belong to the chlamydial pmps, a highly diverse family of autotransporters unique to the *Chlamydia* and ranging from 8 members in *C. trachomatis* to 21 members in *C. pneumoniae*. In addition to the expected *omcA* and *omcB* genes, we detected five adjacent genes sharing a similar N-terminus and conserved cysteine residues, which may form an extended *omc* family, both in *W. chondrophila* and *P. amoebophila*.

Finally, elements of the cell division and peptidoglycan pathways appear to be conserved with *Chlamydiaceae* suggesting that, like its close relatives, *W. chondrophila* uses a FtsZ-independent cell division mechanism (divisome) proposed to involve remnants of peptidoglycan and lipid II pathways [20], [42] (**Figure S7**). Although the gene organization is different, after a detailed structural comparison, we propose that a gene previously annotated in the chlamydial genomes to date as a hypothetical protein might be the missing cell division gene *ftsL*. Moreover, *W. chondrophila* displays additional genes for osmoregulated periplasmic glycan synthesis and cell wall biosynthesis indicating that the cell wall probably differ from other *Chlamydiales*, which will affect host recognition, membrane structure and function.

## Discussion

The full genome sequence of the first representative within the *Waddliaceae* family, *Waddlia chondrophila*, revealed numerous features that provide major insights not only into genome evolution of the order *Chlamydiales* but also into the pathogenic potential of this strict intracellular bacterium. The *Waddlia* genome exhibits numerous repeated sequences and transposases indicating a different genome dynamics from classical *Chlamydia* which almost completely lack repetitive elements. If these repetitions might partially account for *Waddlia’*s larger genome size compared to classical *Chlamydia*, it is then even more interesting that *P. amoebophila,* which has an even larger genome, exhibits fewer repeated elements and reduced metabolic capacities compared to *Waddlia*. Among other features that could explain the increased size of *P. amoebophila* is the F-like DNA conjugative system encoded on a 100 kb-long genomic island [27]. The latter F-like operon is not present in *W. chondrophila* whereas it seems to be at least partially present in another member of the *Parachlamydiaceae* family, *Parachlamydia acanthamoebae* [25], suggesting that it might have been acquired after divergence of *Waddliacaeae* and *Parachlamydiaceae* families.

The *W. chondrophila* plasmid is significantly larger than those of classical *Chlamydia* and encodes few genes with known function. Thanks to its multiple copies, this genetic element might represent an interesting target for diagnostic PCRs of increased sensitivity. Nevertheless, the recent difficulties that appeared with a *C. trachomatis* Swedish variant, where the introduction of a diagnostic PCR targeting the plasmid was rapidly followed by a deletion in the targeted region [43], implies that a multi-target approach should be used. Interestingly, the putative toxin/anti-toxin system indicates that the plasmid might be stably present in the bacteria. Furthermore, the presence of several plasmid regions homologous to the bacterial chromosome suggests that the plasmid might integrate in the genome. This property, if experimentally verified, might open new possibilities for the future development of a genetic manipulation tool.

Another crucial step for the development of a genetic system is the ability to grow the bacteria in a cell-free medium. *W. chondrophila* may represent the best candidate among *Chlamydiales* for axenization, i.e. growth in absence of eukaryotic cells. Indeed, the bacterium possesses all components necessary for the generation of energy and unexpectedly encodes extensive capabilities for *de novo* biosynthesis of essential components such as nucleotides, amino acids, lipids and cofactors. This also suggests that the common ancestor of *Chlamydiales* might have been less dependent upon its eukaryotic host. Different pressures have selected for maintenance or elimination of different metabolic pathways within the different families, compensated by the acquisition of transporters to import the metabolites that cannot be synthesized.

The predicted increased host independence of *W. chondrophila* may partially explain its ability to grow efficiently within a broad host range which includes at least amoebae [44], McCoy cells [10], bovine turbinate cells, P388D1 mouse macrophages [45] and monocyte-derived human macrophages [15]. Moreover, the presence of various mechanisms for pH regulation and defense against radical oxygen species represent key systems for the initial resistance to destruction by professional phagocytes. In macrophages, survival is then achieved by evading the early phagolysosome into a replicative vacuole that forms an intimate association with mitochondria and the endoplasmic reticulum [35]. To modulate and interfere with host functions, the secretion of effectors through a functional type III secretion system is likely essential since a specific inhibition of the T3SS efficiently prevents the growth of *Waddlia* in PBMC-derived human macrophages. The availability of the full genome sequence facilitates the identification of new effectors and virulence factors that will enable us to better understand the mechanisms used by this bacterium to divert the host cell.

The chlamydial outer membrane is unique amongst bacteria and here we demonstrate that not only does *Waddlia* possess OmcA and OmcB, but there is an extended family of OmpA-related proteins which are rich in conserved cysteine clusters and predicted to form outer membrane beta-barrels or porins. Added to this is a predicted outer membrane autotransporter, a putative member of the chlamydial pmp family, that present a homolog in *P. amoebophila* genome. The pmp family members were originally identified as highly immunogenic proteins of the chlamydial cell wall [24] and later shown to be chlamydial adhesins [21], [22]. Further investigations are now needed to confirm the potential roles as adhesins for the putative autotransporters identified. The OmpA family, on the other hand, appears to be confined to the *Chlamydiaceae* and *Waddliaceae*, indicating that its potential role as an adhesin is selective for a common host and tissue range. In this context, it is tempting to speculate that the unique diversity of the OmpA family in the *Waddliaceae* could reflect a wider host specificity as well as a mechanism to avoid immune surveillance, assuming here the role played by the similarly diverse pmps in the *Chlamydiaceae*.

The uniqueness of the *Waddlia* cell wall, combined with what appears to be a family specific complement of proteins well established within the *Chlamydiaceae* to be both highly immunogenic and surface exposed in both the EB and RB forms, raises the exciting prospect that we have identified here prime candidates for serological testing and vaccine development. We can expect that the discovery and isolation of more species within the *Waddliaceae* family will add additional layers of diversity, emphasizing the importance of intensifying investigations into these pathogens.

As a whole, the complete genome sequence of *W. chondrophila* provides new insights into the evolution of the order *Chlamydiales*. The release of further new sequences is now needed to better understand the genetic composition and the genome dynamics of *Chlamydia*-related bacteria. We highlighted the unexpected high biosynthetic capabilities for essential compounds, the presence of several functional virulence factors and discovered large families of outer membrane proteins that might represent good candidates for vaccine development. The availability of the *W. chondrophila* genome will stimulate further research into this medically important bacterial phylum and provides the basis to develop new diagnostic tools which will assist in understanding the pathogenic potential of this bacterium considered as an emerging agent of miscarriage and respiratory tract infections.

## Methods

### Culture and purification of *W. chondrophila*

*Waddlia chondrophila* WSU 86-1044, ATCC number VR-1470, was grown at 32°C within *Acanthamoeba castellanii* ATCC 30010 in 75 cm^2^ cell culture flasks (Becton Dickinson, Franklin Lakes, USA) with 30 ml of peptone-yeast extract glucose broth. To purify *W. chondrophila*, amoebae were removed from culture media using a first centrifugation step at 120×*g* for 10 min. Amoebal debris were next removed from the resuspended bacterial pellet by centrifugation at 6500 x *g* for 30 min onto 25% sucrose (Sigma Aldrich, St Louis, USA) and then at 32000 x *g* for 70 min onto a discontinuous Gastrographin (Bayer Schering Pharma, Zurich, Switzerland) gradient (48%/36%/28%). The bacteria clustering in the Gastrographin gradient at a large lower band were collected, centrifuged at 5800 x *g* and resuspended in PBS twice, and finally stocked at −80°C. The absence of contaminants was confirmed by plating frozen material on Chocolate agar. Since no growth was observed on agar after 72 h of incubation, frozen material was inoculated onto *A. castellanii* and immunofluorescence was performed using specific anti-*Waddlia* antibodies as well as DAPI-staining. We observed no DAPI-positive particles that were not stained with the anti-*Waddlia* antibodies. In addition, a PCR targeting Eubacteria 16S rRNA followed by sequencing was performed with primers FD1 (5′agagtttgatcctggctcag3′) and RP2 (5′acggctaccttgttacgactt3′).

### Genome sequencing, assembly and gap closure

Isolation of genomic DNA from *W. chondrophila* was performed with the Wizard Genomic DNA purification kit (Promega Corporation, Madison, USA). DNA was sequenced using both 454/Roche [46] and Solexa/Illumina [47] technologies, respectively on the Genome Sequencer FLX by Roche Applied Science (Penzberg, Germany) and the Genome Analyzer GAII by Fasteris (Plan les Ouates, Switzerland). GS FLX reads were assembled using Newbler V1.1.02.15 and the 90 large contigs obtained with 40x coverage served as the basis for the gap closure. To scaffold the contigs, a fosmid library with 40 kb DNA inserts was build in the vector pEpiFOS (Epicentre Biotechnologies, Madison, USA) by IIT-Biotech (Bielefeld, Germany). Fosmid walking as well as PCR-based techniques were used to close the gaps. Solexa reads were then mapped to the final assembly with Phrap and visualized with Consed [48]. Thus, 22 homopolymer errors were corrected after manual inspection of discrepancies.

### Genome annotation

Curation and annotation of the genome was performed using the genome annotation system GenDB 2.4 [49]. Prediction of coding sequences (CDS) was accomplished using Critica [50], Glimmer [51] and Reganor [52]. All predicted ORFs were automatically submitted to similarity searches against nr, Swissprot, KEGG, InterPro, Pfam and TIGRfam databases. Putative signal peptides, transmembrane helices and nucleic acid binding domains were predicted using SignalP [53], TMHMM [54] and Helix-Turn-Helix [55], respectively. The automatic annotation of each CDS was manually checked and corrected according to the most congruent tool results. The complete annotated genome sequences have been deposited at GenBank under the accession numbers CP001928 and CP001929.

### Genome analysis

The circular genome plot was created with DNA plotter [56]. Repeats were identified using REPuter [57]. *W. chondrophila* gene content was compared to *P. amoebophila* UWE25 (NC_005861) and *C. trachomatis* D/UW-3/CX (NC_000117) with EDGAR [58], which defines orthologous proteins based on bidirectional best blast hit and then calculates BLASTP score ratio values (SRV). Paralogous genes might be discarded during the analysis. For each comparison, SRV distribution was fitted with binormal or bibeta distribution with a self written R script, and a cutoff was determined at the point where the probability to belong to one or the other peak is equal. Accordingly, a general cutoff of 0.21 was used to retrieve the core genes and singletons.

### Outer membrane proteins analysis

Genes identified as coding for putative outer membrane proteins (omp) and polymorphic membrane proteins (pmp) were aligned using Tcoffee [59] on EMBL-EBI interface (http://www.ebi.ac.uk/Tools/t-coffee/index.html). For omps, a corresponding neighbor-joining tree was calculated using MEGA4 [60] with the following parameters: 1000 bootstrap, pairwise comparison, poisson distribution, gamma parameter equal to 1. The presence of membrane spanning beta-barrel and beta-helical domains were predicted using the programs Partifold [61] and Beta-Wrap Pro [62], respectively.

## Supporting Information

Methods S1 Supplementary methods.
(0.05 MB DOC)

Figure S1 Cumulative GC skew. Representation of the cumulative G toward C bias (G-C) along the genome sequence, which displays the typical « V »-inverted shape. The minimum and maximum of the curves indicate the origin (ori) and terminus (ter) of replication, respectively. dnaA gene positions are indicated by orange dots.
(2.86 MB TIF)

Figure S2 Gene classification according to COG functional categories. (A) The 1934 ORFs of *Waddlia chondrophila* can be classified in several COG functional categories; information storage and processing (orange), cellular processes and signaling (blue), metabolism (green) and poorly characterized (red). No COG could be attributed to 35% of the ORFs (purple). (B) COG classification of genes from *Chlamydia trachomatis* D/UW-3/CX, *Chlamydophila pneumoniae* CWL029, *Protochlamydia amoebophila* UWE25 and *Waddlia chondrophila* WSU 86-1044. The number of genes is shown for categories with more than 1% difference between *W. chondrophila* and *P. amoebophila* or *C. trachomatis*.
(0.55 MB TIF)

Figure S3 Core genes and colinearity. (A) Representation of the number of core genes and singletons of the *Chlamydiales* order as a result of reciprocal best blast hit definition by BLASTP comparison between *Chlamydia trachomatis* D/UW-3C/X, *Protochlamydia amoebophila* UWE25 and *Waddlia chondrophila* WSU 86-1044 using EDGAR software. (B) X-plot of *W. chondrophila* vs. *P. amoebophila*, respectively, *C. trachomatis*. The start position of core genes between two genomes is used to draw a dot, in red, if the genes are on the same strand in both genomes or in blue if the genes are located on opposite strands. Note that *C. trachomatis* genome sequence does not start at the origin of replication.
(0.63 MB TIF)

Figure S4 Type III secretion system of *Chlamydiales* genomes. Position of conserved T3SS genetic clusters spread on the bacterial chromosome in *P. amoebophila* UWE25 (*P.am*), *W. chondrophila* WSU 86-1044 (*W.ch*) and *C. trachomatis* D/UW-3/CX (*C.tr*) from the outermost to the innermost cycle. *C. trachomatis* genome has been rotated to present the putative origin of replication (cumulative GC skew minimum) at position “ori”. Genes encoding for proteins sharing significant amino acid sequence and/or conserved genomic organization are linked by grey shading. Gene names and ORF numbers are listed above and below each gene, respectively. The conserved genes are represented by different colors according to their respective functions. Hypothetical proteins are represented in white and genes encoding for proteins with identified functions likely not involved in T3SS are represented in black. Capital letters refer to sct gene names according to the unified nomenclature proposed by Hueck in 1998. sycE and sycD: genes encoding for SycE-like and SycD/LcrH-like T3SS chaperones. All SycD/LcrH predicted T3SS chaperones contain conserved tetratricopeptide repeats domains (TPRs).
(0.91 MB TIF)

Figure S5 Nucleotide biosynthesis. Schematic representation of the nucleotide biosynthetic pathways and their presence in the different members of the *Chlamydiales* order: all *Chlamydiales* (blue), *P. amoebophila* and *C. trachomatis* (purple), *P. amoebophila* and *W. chondrophila* (green), *W. chondrophila* only (orange). The presence of nucleotide transporters overcomes the lack of *de novo* biosynthetic pathways in *C. trachomatis* and in *P. amoebophila*. *W. chondrophila* exhibits nucleotide transporters, but retains the ability to synthesize pyrimidine from glutamate and possesses only a few genes for the biosynthesis of purine (dashed orange arrow).
(0.55 MB TIF)

Figure S6 *Waddlia* and *Protochlamydia* autotransporters. (A) Schematic representation of classical autotransporter proteins and their representatives in the *Chlamydiales* order. All proteins possess a signal sequence, a passenger domain with functional motives and a C-terminal beta-barrel. (B) BetaWrapPro prediction of beta helix in the putative pmp wcw_0271, a similar prediction is obtained for its homolog in *P. amoebophila* pc0303. The exact structure remains speculative until it can be anchored by biochemical data. Despite the low sequence similarity and differences in size between the *Chlamydiales* pmp members, prediction of similar structural motifs can be obtained. (C) C-terminal alignment between putative pmps of *W. chondrophila* and *P. amoebophila* showing a more conserved region predicted to encode a 16-pass beta-barrel by Partifold software.
(1.21 MB TIF)

Figure S7 Peptidoglycan and proteins involved in cell division. Schematic comparison of the divisome of *E. coli* with *W. chondrophila*. The approximate topologies and localizations of selected cell divisome proteins, established for *E. coli*, are shown in the left panel (A) and the postulated remnant divisome of *W. chondrophila* in the right panel (B). Orientation is with the outer membrane (OM) with liposaccharide uppermost, and the cytosolic side of the inner membrane (IM) below. The peptidoglycan layer (PG) in the periplasmic space includes glycosyl-crosslinks (red bars) in A, which are thought to be absent in members of the *Chlamydiales* order (B). Indeed, there is no convincing chemical evidence for the presence of peptidoglycan in *Chlamydia*, despite the retention of the genes involved in peptidoglycan metabolism (McCoy & Maurelli 2006). Transmembrane helices of membrane proteins are represented by cylinders. The most notable absentee in all chlamydial genomes to date, including *W. chondrophila*, is the tubulin homolog FtsZ, which occupies a central role in forming and localizing the septal ring in the majority of bacteria. All members of the *Chlamydiales* remnant divisome are essential components of late stage septal peptidoglycan synthesis (see McCoy & Maurelli 2006, Blaauwen 2008, Vollmer & Bertsche 2008, Henrichfreise 2009), raising the possibility that this function has been retained in *W. chondrophila*.
(6.40 MB TIF)

Table S1 Repartition of COG categories in various *Chlamydiales* genomes. Number and percentage of genes in different COG categories as extracted from genome annotation (*W. chondrophila*) or NCBI genome repository (*C. trachomatis*, *Cp. pneumoniae*, *P. amoebophila*).
(0.05 MB DOC)

Table S2 Ability to synthesize amino acids. The ability to synthesize the various amino acids is reported here for *C. trachomatis*, *P. amoebophila* and *W. chondrophila* as inferred from the analysis of KEGG pathways.
(0.04 MB DOC)
