# Supplementary material for: The Waddlia Genome: A Window into Chlamydial Biology
Source: PLoS One. 2010 May 28;5(5):e10890. doi: 10.1371/journal.pone.0010890 (PMC2878342; doi:10.1371/journal.pone.0010890)
Supplement: Methods S1 — Supplementary methods. (0.05 MB DOC) [file pone.0010890.s001.doc]

**SUPPLEMENTARY DATA**

SUPPLEMENTARY METHODS

**GC skew analysis**

The GC skew measures the excess of Gs by calculating the difference between the number of Gs and Cs (G-C) in a sliding window of 1000 nucleotides. The skews were cumulated to obtain the cumulative GC skew that represents the sum of the GC skews from the first to the i^th^ window.

**Infection of PBMC-derived human macrophages**

**Bacterial strain.** *W. chondrophila* strain WSU 86-10440 was grown at 32 °C within *Acanthamoeba castellanii* in 75-cm^2^ cell culture flasks (Corning, New-York, USA) with 30 ml of peptone-yeast extract-glucose broth [1]. After 6 days of incubation, cultures were harvested and the broth was filtered through a 5μm pore to eliminate both amoebal trophozoites and cysts and to collect bacteria in the flow through.

**Antibodies, probes and reagents.** Bacteria were detected by immunofluorescence using in-house polyclonal mouse or rabbit anti-*Waddlia* antibodies. In-house polyclonal antibodies were generated as follows: rabbits and mice were inoculated 4 times at day 0, 14, 28 and 56 with 500 μl of PBS containing 5x10^8^ of heat-inactivated *Waddlia*. Bleedings were obtained before immunization (pre-immune sera) and at day 90.

The secondary antibodies used were: Alexa Fluor 488 donkey anti-rabbit, Alexa Fluor 488 goat anti-mouse (Molecular Probes, OR, USA). Concanavalin A (Molecular Probes, OR, USA) was used to stain macrophages. T3SS inhibitors ME0052 and ME0053 were kindly provided by Dr. Elofsson (Department of chemistry, Umeå University, Sweden). These compounds have been described previously using different names: ME0052 is identical to INP0010 and compound 8 in [2]; ME0053 is identical to INP0403 and compound 11 in [2]. Stock solutions of 10mM in DMSO were prepared and infected PBMC-derived human macrophages were treated with the final concentrations of inhibitors indicated. The final DMSO concentration was kept below 0.5%.

**Macrophages**. Blood from different healthy volunteers was collected in tubes containing EDTA as an anticoagulant. Peripheral blood mononuclear cells were diluted in 0.9% NaCl and were separated by centrifugation at 750 × g for 20 min on Ficoll (Eurobio, Les Ulis, France) and suspended in RPMI supplemented with 200 mM L-glutamine (Gibco-BRL, Life Technologies, Paisley, Scotland) and 10% fetal calf serum (Gibco-BRL). Then, 10^5^ peripheral blood mononuclear cells/ml were incubated for 1h at 37 °C in 24-well cell culture plates (Corning). Non-adherent cells were washed and the remaining adherent cells were considered monocytes since more than 95% of these cells expressed CD14 [3,4]. Monocytes were further differentiated into macrophages by incubation at 37 °C for 7 days in the presence of fetal calf serum.

**Infection procedure.** The relative bacterial concentration was determined by staining the bacteria with the LIVE/DEAD BacLight Bacterial Viability Kits (Molecular Probes) and was used to estimate the dilution factor to apply to the bacterial culture to infect the macrophages with an MOI between 1 and 10. Plates were centrifuged at 1790 × *g* for 10 min at room temperature. After 15 min of incubation at 37 °C, cells were washed with RPMI+10% FCS and further incubated for different periods at 37 °C.

**Confocal microscopy.** Infected macrophages were washed with PBS, fixed with ice-cold methanol for 4 min. Cells were then washed three times with PBS and were then blocked and permeabilised for 1h in a blocking solution (PBS/0.1% saponin/1% bovine serum albumin (BSA)). Saponin (0.1%) was included in all subsequent incubation steps. Coverslips were incubated with primary antibodies directed against bacteria for 1h at room temperature in blocking solution. After washing three times with PBS/0.1% saponin, coverslips were incubated for 1h with secondary antibodies and concanavalin A in blocking solution. After washing three times with PBS and once with deionised water, the coverslips were mounted onto glass slides using Mowiol (Sigma-Aldrich, MO, USA). Cells were observed on a confocal fluorescence microscope (Zeiss LSM 510 Meta, Jena, Germany). Files were analysed using Adobe Photoshop and Image J for microscopy softwares ([www.macbiophotonics.com](http://www.macbiophotonics.com/)). The number of bacteria per macrophage was determined by counting the number of bacteria within about 100 macrophages. All the assays were performed at least in triplicate.

REFERENCES

1. Greub G, Raoult D (2002) Crescent bodies of Parachlamydia acanthamoeba and its life cycle within Acanthamoeba polyphaga: an electron micrograph study. Appl Environ Microbiol 68: 3076-3084.

2. Nordfelth R, Kauppi AM, Norberg HA, Wolf-Watz H, Elofsson M (2005) Small-molecule inhibitors specifically targeting type III secretion. Infect Immun 73: 3104-3114.

3. Mege JL, Sanguedolce MV, Jacob T, Bongrand P, Capo C, et al. (1993) Nocardia fractions, NLD and NWSM, induce tumor necrosis factor-alpha secretion in human monocytes: role of protein kinase C. Eur J Immunol 23: 1582-1587.

4. Capo C, Zugun F, Stein A, Tardei G, Lepidi H, et al. (1996) Upregulation of tumor necrosis factor alpha and interleukin-1 beta in Q fever endocarditis. Infect Immun 64: 1638-1642.
