# Supplementary figures and images for: The Waddlia Genome: A Window into Chlamydial Biology
Source: PLoS One. 2010 May 28;5(5):e10890. doi: 10.1371/journal.pone.0010890 (PMC2878342; doi:10.1371/journal.pone.0010890)

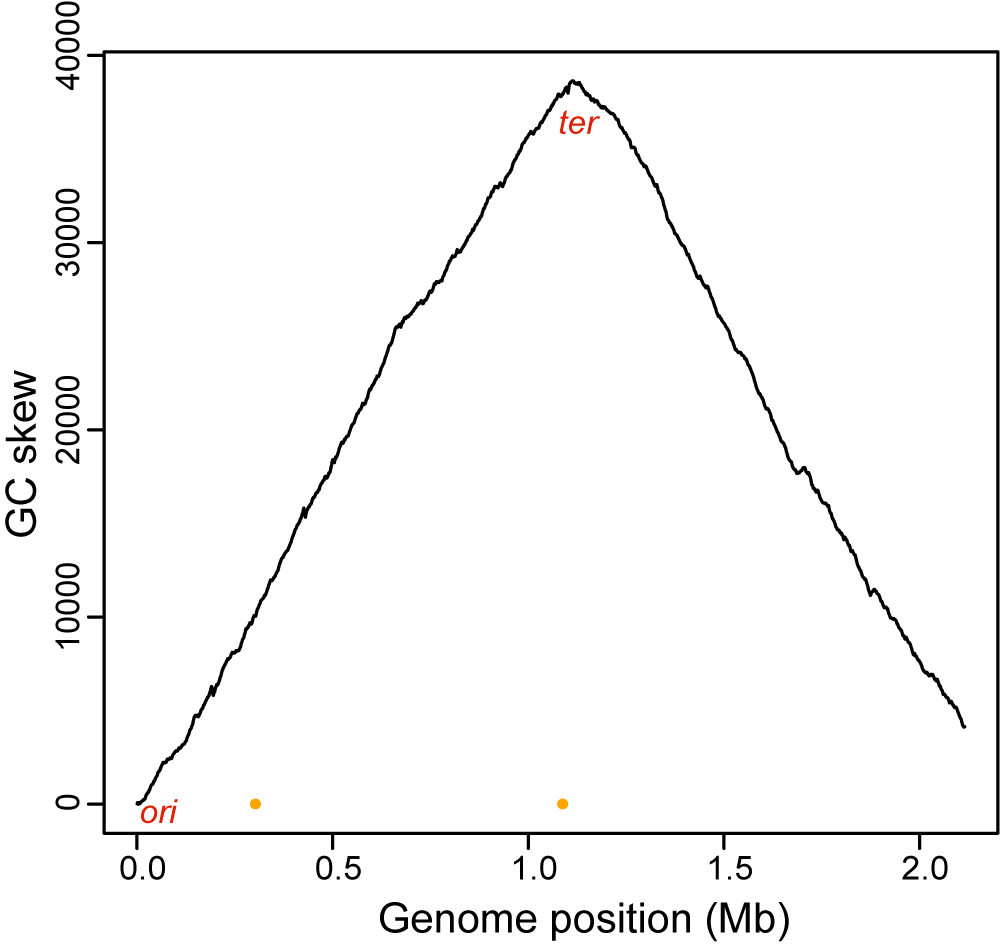

Supplement: Figure S1 — Cumulative GC skew. Representation of the cumulative G toward C bias (G-C) along the genome sequence, which displays the typical « V »-inverted shape. The minimum and maximum of the curves indicate the origin (ori) and terminus (ter) of replication, respectively. dnaA gene positions are indicated by orange dots. (2.86 MB TIF) [file pone.0010890.s002.tif]

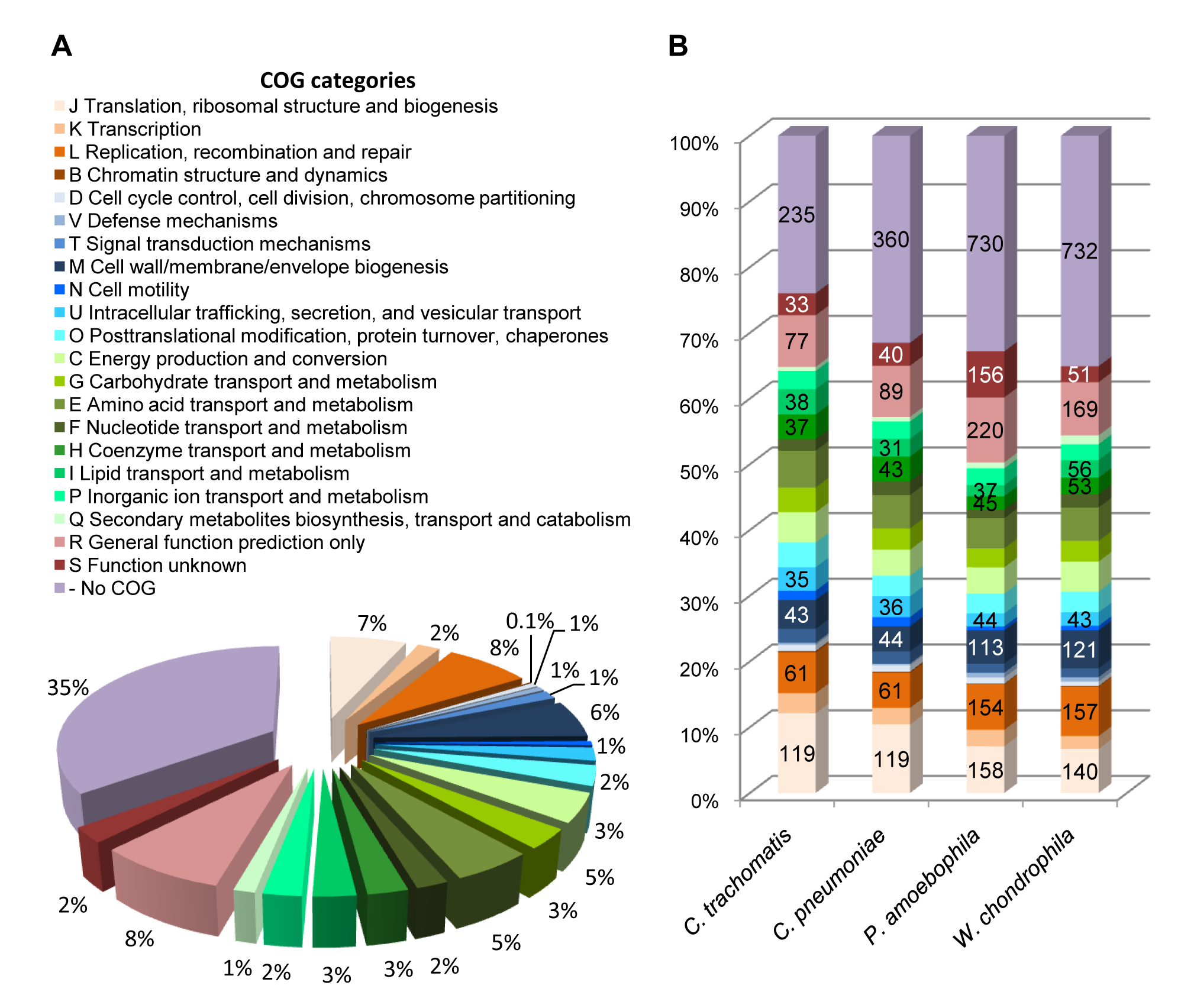

Supplement: Figure S2 — Gene classification according to COG functional categories. (A) The 1934 ORFs of Waddlia chondrophila can be classified in several COG functional categories; information storage and processing (orange), cellular processes and signaling (blue), metabolism (green) and poorly characterized (red). No COG could be attributed to 35% of the ORFs (purple). (B) COG classification of genes from Chlamydia trachomatis D/UW-3/CX, Chlamydophila pneumoniae CWL029, Protochlamydia amoebophila UWE25 and Waddlia chondrophila WSU 86-1044. The number of genes is shown for categories with more than 1% difference between W. chondrophila and P. amoebophila or C. trachomatis. (0.55 MB TIF) [file pone.0010890.s003.tif]

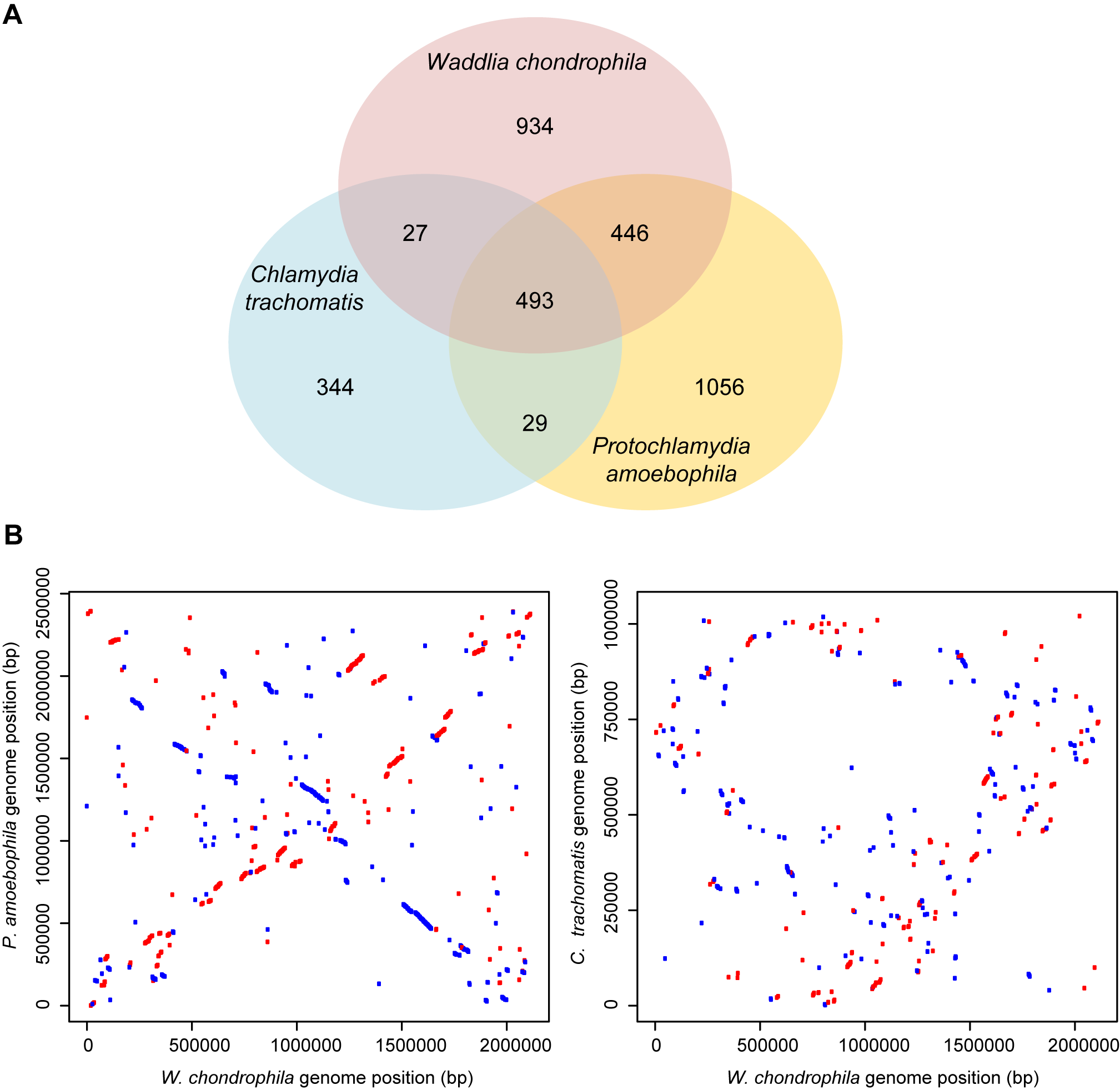

Supplement: Figure S3 — Core genes and colinearity. (A) Representation of the number of core genes and singletons of the Chlamydiales order as a result of reciprocal best blast hit definition by BLASTP comparison between Chlamydia trachomatis D/UW-3C/X, Protochlamydia amoebophila UWE25 and Waddlia chondrophila WSU 86-1044 using EDGAR software. (B) X-plot of W. chondrophila vs. P. amoebophila, respectively, C. trachomatis. The start position of core genes between two genomes is used to draw a dot, in red, if the genes are on the same strand in both genomes or in blue if the genes are located on opposite strands. Note that C. trachomatis genome sequence does not start at the origin of replication. (0.63 MB TIF) [file pone.0010890.s004.tif]

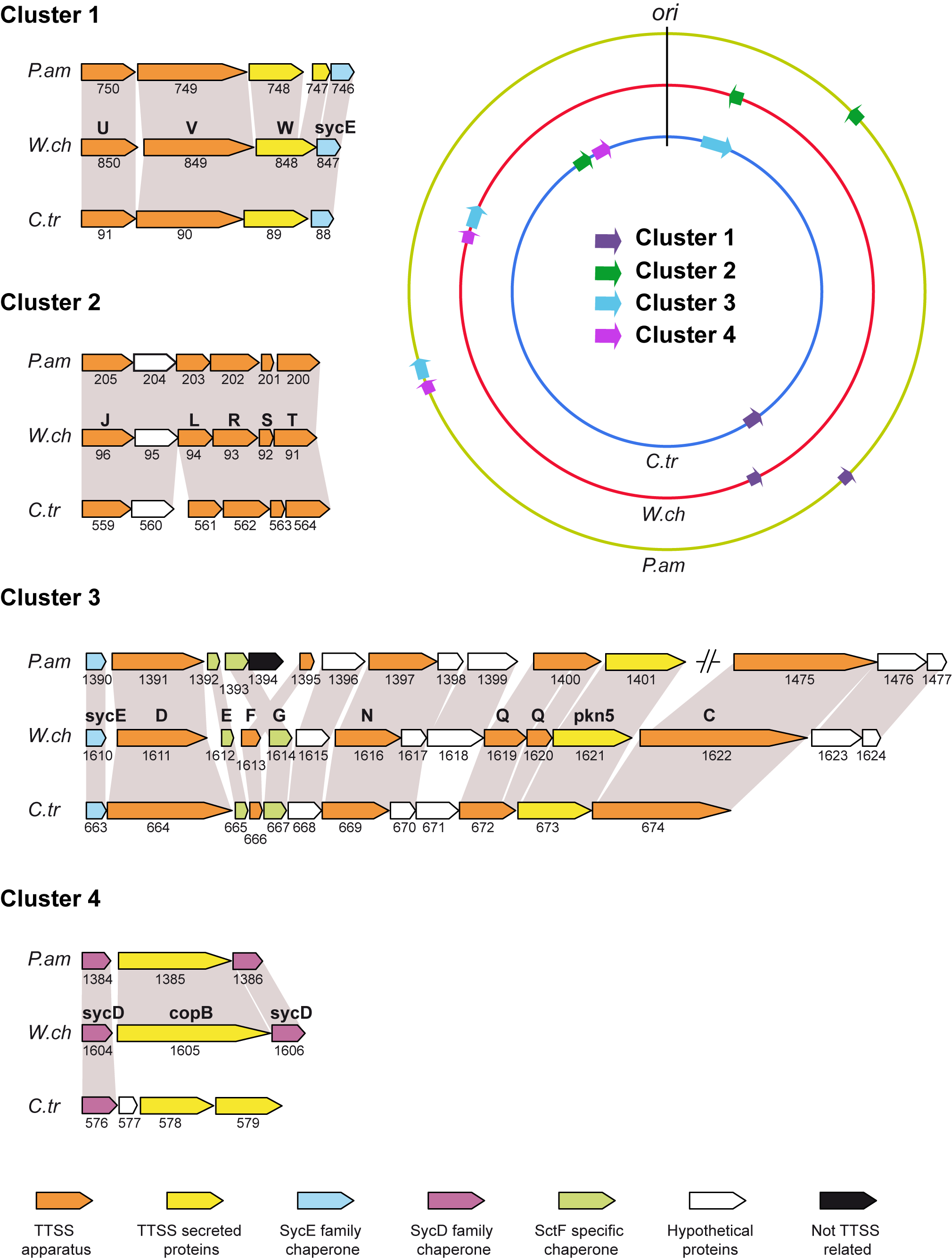

Supplement: Figure S4 — Type III secretion system of Chlamydiales genomes. Position of conserved T3SS genetic clusters spread on the bacterial chromosome in P. amoebophila UWE25 (P.am), W. chondrophila WSU 86-1044 (W.ch) and C. trachomatis D/UW-3/CX (C.tr) from the outermost to the innermost cycle. C. trachomatis genome has been rotated to present the putative origin of replication (cumulative GC skew minimum) at position “ori”. Genes encoding for proteins sharing significant amino acid sequence and/or conserved genomic organization are linked by grey shading. Gene names and ORF numbers are listed above and below each gene, respectively. The conserved genes are represented by different colors according to their respective functions. Hypothetical proteins are represented in white and genes encoding for proteins with identified functions likely not involved in T3SS are represented in black. Capital letters refer to sct gene names according to the unified nomenclature proposed by Hueck in 1998. sycE and sycD: genes encoding for SycE-like and SycD/LcrH-like T3SS chaperones. All SycD/LcrH predicted T3SS chaperones contain conserved tetratricopeptide repeats domains (TPRs). (0.91 MB TIF) [file pone.0010890.s005.tif]

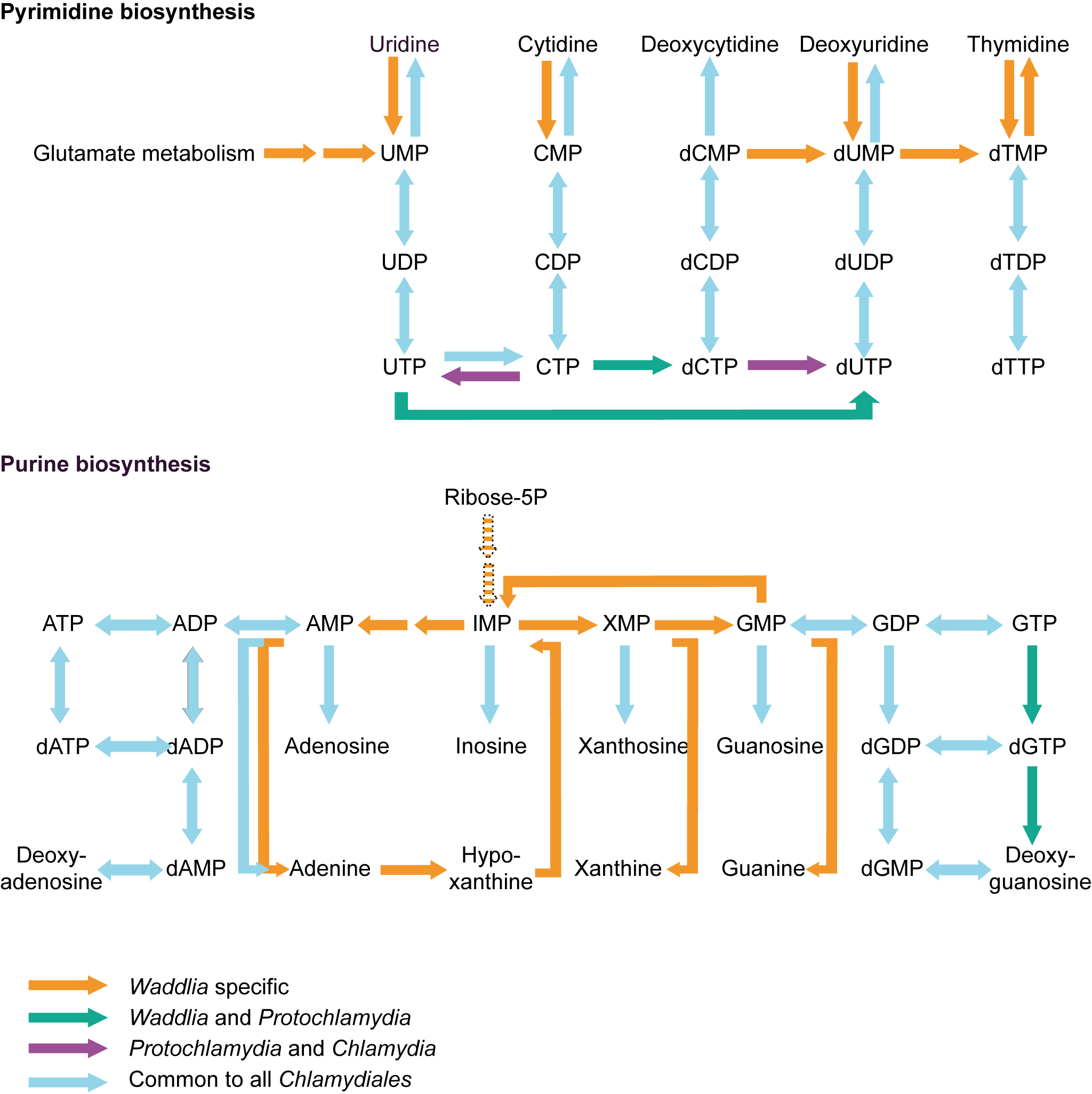

Supplement: Figure S5 — Nucleotide biosynthesis. Schematic representation of the nucleotide biosynthetic pathways and their presence in the different members of the Chlamydiales order: all Chlamydiales (blue), P. amoebophila and C. trachomatis (purple), P. amoebophila and W. chondrophila (green), W. chondrophila only (orange). The presence of nucleotide transporters overcomes the lack of de novo biosynthetic pathways in C. trachomatis and in P. amoebophila. W. chondrophila exhibits nucleotide transporters, but retains the ability to synthesize pyrimidine from glutamate and possesses only a few genes for the biosynthesis of purine (dashed orange arrow). (0.55 MB TIF) [file pone.0010890.s006.tif]

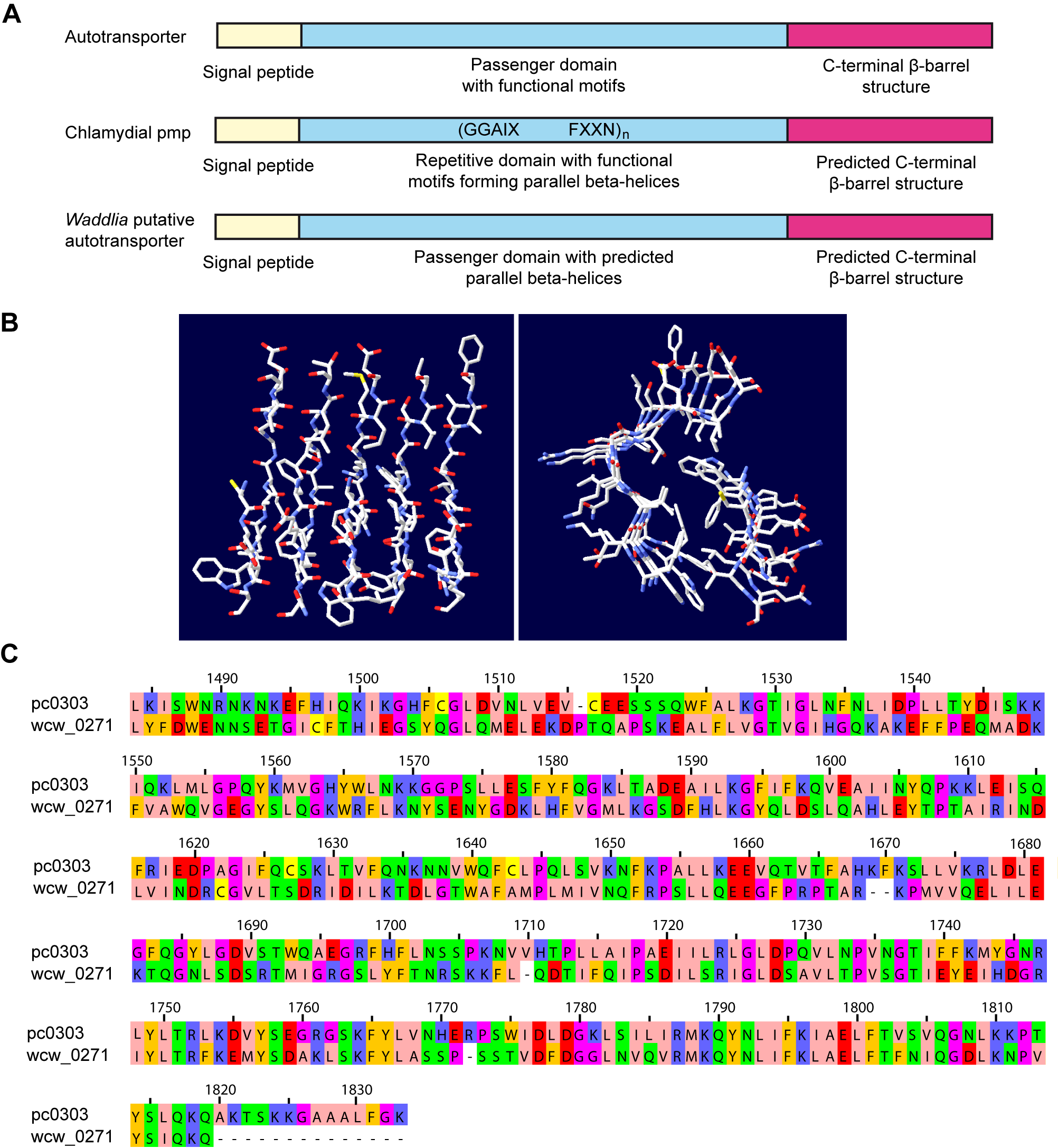

Supplement: Figure S6 — Waddlia and Protochlamydia autotransporters. (A) Schematic representation of classical autotransporter proteins and their representatives in the Chlamydiales order. All proteins possess a signal sequence, a passenger domain with functional motives and a C-terminal beta-barrel. (B) BetaWrapPro prediction of beta helix in the putative pmp wcw_0271, a similar prediction is obtained for its homolog in P. amoebophila pc0303. The exact structure remains speculative until it can be anchored by biochemical data. Despite the low sequence similarity and differences in size between the Chlamydiales pmp members, prediction of similar structural motifs can be obtained. (C) C-terminal alignment between putative pmps of W. chondrophila and P. amoebophila showing a more conserved region predicted to encode a 16-pass beta-barrel by Partifold software. (1.21 MB TIF) [file pone.0010890.s007.tif]

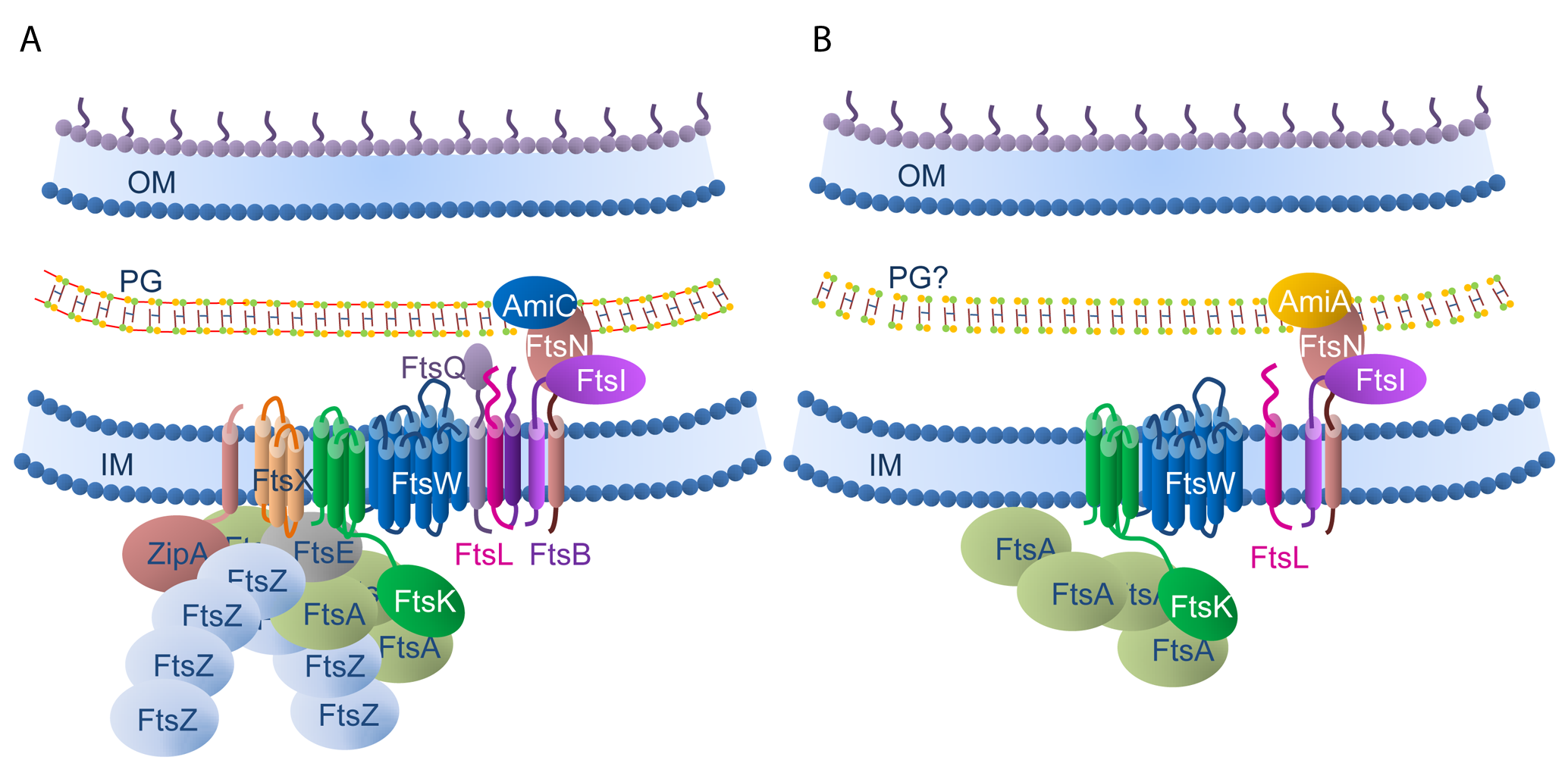

Supplement: Figure S7 — Peptidoglycan and proteins involved in cell division. Schematic comparison of the divisome of E. coli with W. chondrophila. The approximate topologies and localizations of selected cell divisome proteins, established for E. coli, are shown in the left panel (A) and the postulated remnant divisome of W. chondrophila in the right panel (B). Orientation is with the outer membrane (OM) with liposaccharide uppermost, and the cytosolic side of the inner membrane (IM) below. The peptidoglycan layer (PG) in the periplasmic space includes glycosyl-crosslinks (red bars) in A, which are thought to be absent in members of the Chlamydiales order (B). Indeed, there is no convincing chemical evidence for the presence of peptidoglycan in Chlamydia, despite the retention of the genes involved in peptidoglycan metabolism (McCoy & Maurelli 2006). Transmembrane helices of membrane proteins are represented by cylinders. The most notable absentee in all chlamydial genomes to date, including W. chondrophila, is the tubulin homolog FtsZ, which occupies a central role in forming and localizing the septal ring in the majority of bacteria. All members of the Chlamydiales remnant divisome are essential components of late stage septal peptidoglycan synthesis (see McCoy & Maurelli 2006, Blaauwen 2008, Vollmer & Bertsche 2008, Henrichfreise 2009), raising the possibility that this function has been retained in W. chondrophila. (6.40 MB TIF) [file pone.0010890.s008.tif]
