# Supplementary material for: The Waddlia Genome: A Window into Chlamydial Biology
Source: PLoS One. 2010 May 28;5(5):e10890. doi: 10.1371/journal.pone.0010890 (PMC2878342; doi:10.1371/journal.pone.0010890)
Supplement: Table S1 — Repartition of COG categories in various Chlamydiales genomes. Number and percentage of genes in different COG categories as extracted from genome annotation (W. chondrophila) or NCBI genome repository (C. trachomatis, Cp. pneumoniae, P. amoebophila). (0.05 MB DOC) [file pone.0010890.s009.doc]

| Code | Description | *Chlamydia trachomatis* D/UW-3/CX | *Chlamydophila pneumoniae* CWL029 | *Protochlamydia amoebophila* UWE25 | *Waddlia chondrophila*  WSU 86-1044 |
| --- | --- | --- | --- | --- | --- |
| J | Translation, ribosomal structure and biogenesis | 119 (12.13) | 119 (10.41) | 158 (7.10) | 140 (6.70) |
| K | Transcription | 30 (3.05) | 29 (2.53) | 56 (2.51) | 41 (1.96) |
| L | Replication, recombination and repair | 61 (6.21) | 61 (5.33) | 154 (6.92) | 157 (7.51) |
| B | Chromatin structure and dynamics | 2 (0.20) | 2 (0.17) | 2 (0.08) | 2 (0.09) |
| D | Cell cycle control, cell division, chromosome partitioning | 9 (0.91) | 11 (0.96) | 21 (0.94) | 14 (0.67) |
| V | Defense mechanisms | 3 (0.30) | 3 (0.26) | 16 (0.71) | 14 (0.67) |
| T | Signal transduction mechanisms | 21 (2.14) | 21 (1.83) | 31 (1.39) | 28 (1.34) |
| M | Cell wall/membrane/ envelope biogenesis | 43 (4.38) | 44 (3.84) | 113 (5.07) | 121 (5.79) |
| N | Cell motility | 14 (1.42) | 16 (1.39) | 13 (0.58) | 14 (0.67) |
| U | Intracellular trafficking, secretion, and vesicular transport | 35 (3.56) | 36 (3.14) | 44 (1.97) | 43 (2.05) |
| O | Posttranslational modification, protein turnover, chaperones | 37 (3.77) | 36 (3.14) | 67 (3.01) | 66 (3.16) |
| C | Energy production and conversion | 45 (4.58) | 45 (3.93) | 89 (4) | 95 (4.54) |
| G | Carbohydrate transport and metabolism | 37 (3.77) | 37 (3.23) | 64 (2.87) | 66 (3.16) |
| E | Amino acid transport and metabolism | 55 (5.60) | 58 (5.07) | 102 (4.58) | 106 (5.07) |
| F | Nucleotide transport and metabolism | 17 (1.73) | 24 (2.09) | 30 (1.34) | 42 (2.01) |
| H | Coenzyme transport and metabolism | 37 (3.77) | 43 (3.76) | 45 (2.02) | 53 (2.53) |
| I | Lipid transport and metabolism | 38 (3.87) | 31 (2.71) | 37 (1.66) | 56 (2.68) |
| P | Inorganic ion transport and metabolism | 27 (2.75) | 30 (2.62) | 57 (2.56) | 50 (2.39) |
| Q | Secondary metabolites biosynthesis, transport and catabolism | 6 (0.61) | 8 (0.69) | 20 (0.89) | 28 (1.34) |
| R | General function prediction only | 77 (7.84) | 89 (7.78) | 220 (9.88) | 169 (8.09) |
| S | Function unknown | 33 (3.36) | 40 (3.49) | 156 (7.01) | 51 (2.44) |
| - | Not in COGs | 235 (23.95) | 360 (31.49) | 730 (32.80) | 732 (35.05) |
