# Supplementary material for: The Waddlia Genome: A Window into Chlamydial Biology
Source: PLoS One. 2010 May 28;5(5):e10890. doi: 10.1371/journal.pone.0010890 (PMC2878342; doi:10.1371/journal.pone.0010890)
Supplement: Table S2 — Ability to synthesize amino acids. The ability to synthesize the various amino acids is reported here for C. trachomatis, P. amoebophila and W. chondrophila as inferred from the analysis of KEGG pathways. (0.04 MB DOC) [file pone.0010890.s010.doc]

| Amino acid | *Chlamydia trachomatis* D/UW-3/CX | *Protochlamydia amoebophila* UWE25 | *Waddlia chondrophila*  WSU 86-1044 |
| --- | --- | --- | --- |
| Alanine | no | yes | yes |
| Arginine | no | no | no |
| Asparagine | no | no | yes |
| Aspartate | yes | yes | yes |
| Cysteine | no | no | yes |
| Glutamate | no | yes | yes |
| Glutamine | no | yes | yes |
| Glycine | yes | yes | yes |
| Histidine | no | no | no |
| Isoleucine | no | no | no |
| Leucine | no | no | no |
| Lysine | no^1^ | no^1^ | no^1^ |
| Methionine | no | no | yes^2^ |
| Phenylalanine | no | no | no |
| Proline | no | yes | yes |
| Serine | no | yes | yes |
| Threonine | no | yes | yes |
| Tryptophan | yes | no | no |
| Tyrosine | no | no | no |
| Valine | no | no | no |
| ^1^ Although the pathway for lysine biosynthesis is almost complete, the last enzyme (LysA) is missing rending its synthesis hypothetical. | | | |
| ^2^  *W. chondrophila* likely synthesizes methionine from cysteine since most genes could be annotated; however one enzyme could not be precisely identified. | | | |
